# Primary Pterygium Excision Surgery: Analysis of Risk Factors and Clinical Outcomes

**DOI:** 10.7759/cureus.62440

**Published:** 2024-06-15

**Authors:** Kirupakaran Arun, Fiorella Grillon, Panagiotis Georgoudis

**Affiliations:** 1 Ophthalmology, Whipps Cross Hospital, London, GBR

**Keywords:** amniotic membrane transplantation, conjunctival limbal autograft, pterygium recurrence, primary pterygium excision, pterygium excision

## Abstract

Purpose

To evaluate clinical outcomes of primary pterygium excision surgery and analyze risk factors for pterygium recurrence.

Setting

Eye Treatment Centre, Cornea and External Diseases Service, Whipps Cross Hospital, London, United Kingdom.

Methods

Retrospective case series of eyes undergoing primary pterygium excision between August 2017 and July 2022. Patients who underwent “pterygium excision” documented in the electronic patient record system were identified. Patients with recurrent pterygium and those lost-to-follow-up were excluded. The duration of follow-up, type of surgery performed (primary conjunctival closure, conjunctival autograft, and amniotic membrane transplantation), recurrences with respect to the type of surgery performed, and postoperative complications were collected and analyzed.

Results

In total, 83 eyes (from 79 patients) were included. The mean age of our patient cohort was 59.3 ± 5.9 years. The most common ethnic distribution was Black Caribbean (15.7%). Conjunctival autograft was performed in 76 eyes (91.6%), primary conjunctival closure was performed in five eyes (6%) and amniotic membrane transplantation was performed in two eyes (2.4%). The recurrence rate with conjunctival autograft was 1.3% with a median time to recurrence of 2.98 months. Recurrence was significantly more common in patients below the age of 40 years (p=0.03). Recurrence was not significantly associated with gender (p=0.23), ethnicity (p=0.17), or grade of surgeon (p=0.38).

Conclusion

Our findings demonstrate the effectiveness of conjunctival autograft with fibrin glue fixation for the surgical management of primary pterygium. Recurrence was found to be significantly more common in patients under the age of 40 years old. However, recurrence was not associated with ethnicity, gender, or surgeon grade.

## Introduction

A pterygium is a wing-shaped fibrovascular growth of the bulbar conjunctiva that can extend onto the cornea. Chronic exposure to ultraviolet light has been implicated as the biggest risk factor and explains the variable incidence of pterygium (1-15%) depending on geographic location [1-3]. In some patients, it can induce symptoms of intermittent redness, foreign body sensation, and cause refractive errors from induced astigmatism [4].

Even though medical treatment can relieve symptoms of redness and irritation, surgery remains the treatment of choice to eliminate the pterygium and restore the integrity of the ocular surface. The indications for surgery include chronic irritation and inflammation, cosmesis, restriction of eye movements, and reduced vision due to irregular astigmatism or encroachment of the visual axis [5].

There are several surgical techniques for pterygium excision with variable reported recurrence rates. The classical technique was the bare sclera approach which involves simple pterygium excision and is associated with a high recurrence rate of 24-89% [6-7]. Pterygium excision with the use of anti-fibrotic agents such as mitomycin-C or beta irradiation can decrease the recurrence rate to 10-24% but is associated with serious side effects including scleral melt [8, 9].

As of today, conjunctival autograft (CAG) transplantation is considered the gold-standard approach for pterygium excision due to its low recurrence rates of 2-16.7% [10]. It involves the placement of conjunctiva from another part of the eye over the bare scleral defect left following pterygium excision. Different methods of graft fixation have been reported such as fibrin glue, sutures, and electrocauterization [11, 12]. 

Amniotic membrane transplantation (AMT) has been used as an alternative to CAG to avoid any disruption to areas of healthy conjunctiva, but this has shown to be inferior at reducing recurrence compared to CAG with recurrence rates of 4.8-26.9% [12].

Our study aimed to evaluate the results of primary pterygium excision at an NHS UK hospital setting and analyze for risk factors that can affect the recurrence rate.

## Materials and methods

This study was an electronic retrospective case review of eyes that underwent primary pterygium excision with conjunctival autograft over 60 months during the period 1^st^ August 2017 to 31^st^ July 2022. The audit was approved by the Whipps Cross Hospital Audit Department and adhered to the tenets of the Declaration of Helsinki and the UK Data Protection Act (2018). 

Case ascertainment

The audit suite of our hospital’s electronic patient record system (Medisoft) was used to identify all patients were underwent pterygium excision procedures at our unit. The electronic patient records of these patients were reviewed to ascertain whether the individual met the case definition for inclusion in the study.

Case definition

The inclusion criteria included primary pterygium and corneal invasion of > 2 mm. Patients were excluded based on the following criteria: previous pterygium excision surgery, active ocular surface inflammation, and patients lost to follow-up within 12 months of surgery. The patient demographics, duration of follow-up, surgeon grade, recurrence rate, and intra or postoperative complications were analyzed.

Surgical technique

**Figure 1:**
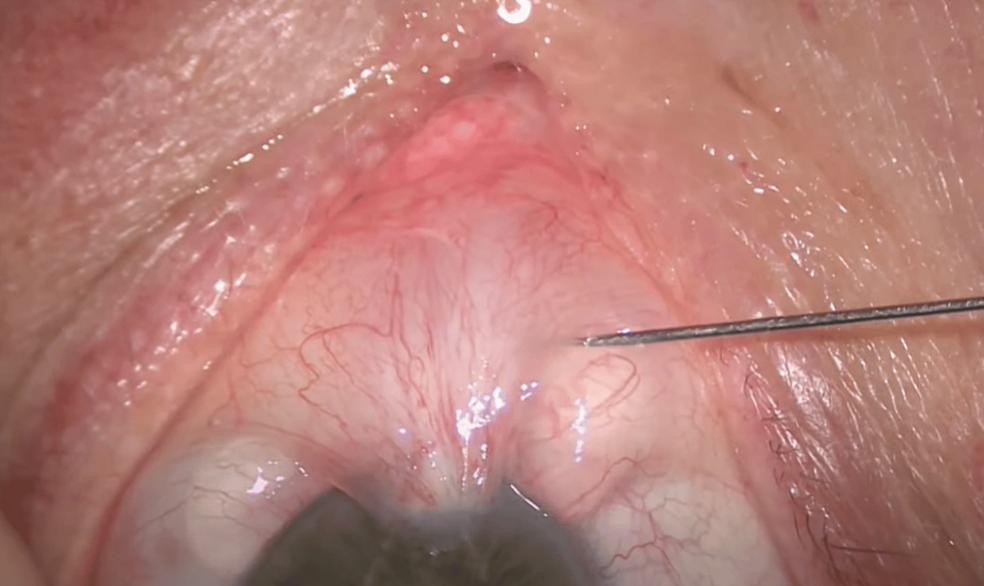
Injection of 2% lignocaine into the body of the pterygium Intra-operative photograph using the same technique. This figure is published with permission from Uday Devgan, MD, of CataractCoach.com [13].

All surgeries were performed by a single corneal consultant or by an ophthalmology resident supervised by the same corneal consultant. The surgical technique followed for each procedure is described below. All patients were anaesthetized with topical anesthesia (proxymetacaine hydrochloride 0.5% and tetracaine hydrochloride 0.5%) and subconjunctival 2% lignocaine hydrochloride into the body of the pterygium (Figure 1).

**Figure 2:**
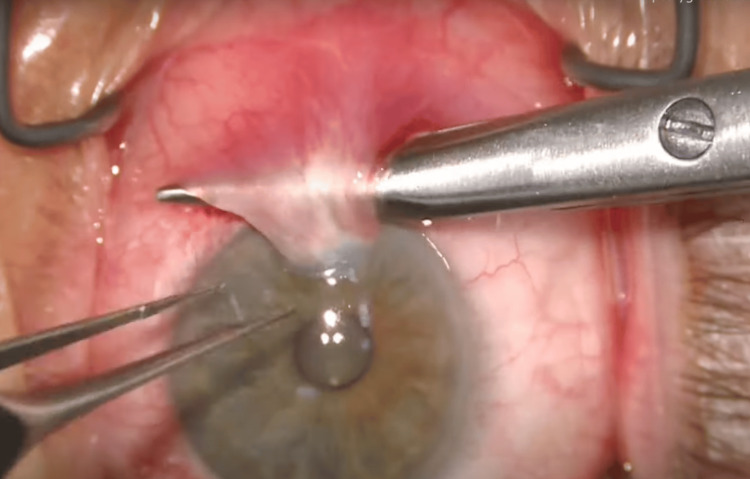
Westcott scissors are used to create a pocket under the pterygium Intra-operative photograph using the same technique. This figure is published with permission from Uday Devgan, MD, of CataractCoach.com [13].

The superior and inferior parts of the pterygium were bluntly dissected away from the sclera using Wescott scissors to create a pocket under the pterygium (Figure 2).

Artery clips were clipped to the pterygium and then rotated away from the cornea to help peel the head of the pterygium from the cornea. The artery clips were then removed and the corneal and limbal surfaces were smoothed by scraping with a crescent blade. The pterygium was then cut from its base and sent for histopathology. A tenonectomy was then performed with extra care taken to avoid damage to the underlying muscles. 

Primary Closure

Relieving incisions were performed to mobilize the limbal conjunctiva and sutured with 8/0 vicryl to cover the bare sclera.

Conjunctival Autograft

**Figure 3:**
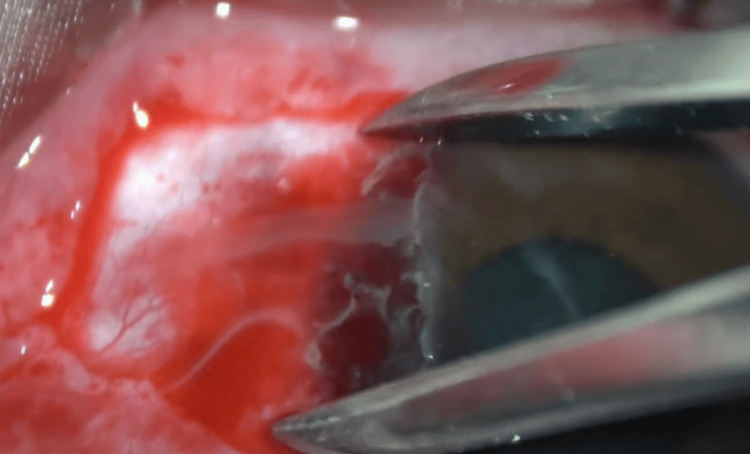
Calipers are used to measure the maximum area of bare scleral bed left following pterygium excision Intra-operative photograph using the same technique. This figure is published with permission from Uday Devgan, MD, of CataractCoach.com [14].

The bare scleral bed was measured in a position with the eye deviated to the opposite side of the pterygium to obtain the maximum area of defect (Figure 3).

**Figure 4:**
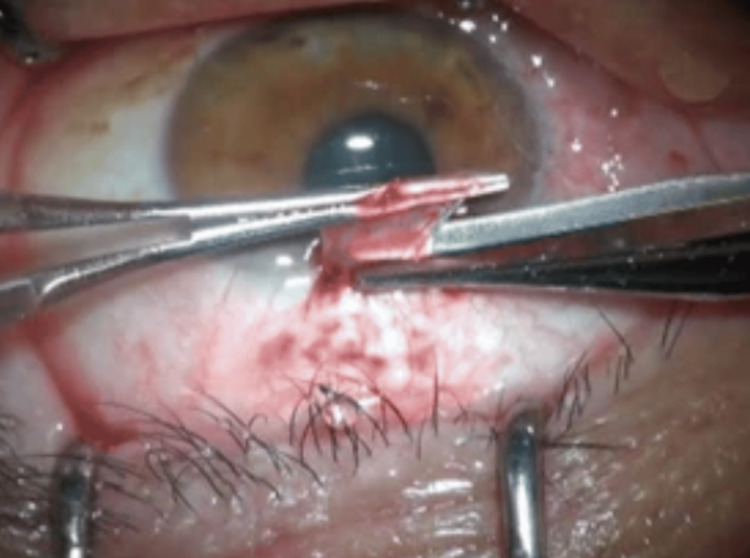
Superior conjunctival autograft is dissected Intra-operative photograph from the same technique. This figure is published with permission from Uday Devgan, MD, of CataractCoach.com [14].

The eyeball is then rotated downwards and an area of the superior bulbar conjunctiva adjacent to the limbus was demarcated. The most common size used was 5 mm x 9 mm. Blunt dissection of the conjunctiva was carried out with the underlying Tenon‘s capsule (Figure 4).

The autograft was then slid into place over the bare sclera in the correct anatomical orientation (limbal side of the graft to the limbal side of the defect) and secured into position with fibrin glue (Tisseel - Baxter International).

Amniotic Membrane Transplantation

The amniotic membrane was removed from the nitrocellulose paper placed over the bare sclera (with the epithelial side up) and secured in place with Tisseel glue. Any excess amniotic membrane was trimmed.

Post-operative care and follow-up

Post-operatively, all patients were started on topical steroids (tapered over three months) and topical chloramphenicol for two weeks. Patients were followed up after one month, three months, six months, and annually thereafter.

We classified recurrence as fibrovascular growth seen at the site of the previous pterygium excision that was crossing the limbus.

Statistical analysis

All data were collected and analyzed using Microsoft Excel (Microsoft Corporation, Redmond, WA) and GraphPad Prism version 8.0.0 for Mac (GraphPad Software, San Diego, California USA). Quantitative data were calculated as mean and standard deviation. Qualitative data were calculated into frequency. A multivariate logistic regression model (Chi-squared test) was used to determine factors associated with pterygium recurrence. Statistical significance was set at p < 0.05. 

## Results

Demographic data 

Between 1^st^ August 2017 to 31^st^ July 2022, 103 eyes underwent pterygium excision. Of these, 83 eyes (from 79 patients) met the study inclusion criteria. 

A summary of the demographic data is shown in Table 1. The mean age of patients was 59.3 years (range 31 to 79 years) and 44/83 (53.0%) of patients were female. The three most common patient ethnic backgrounds were Black Caribbean (15.7%), Black African (12.0%) and Indian (12.0%). The mean length of follow-up was 29 months (range 12 to 67 months).

**Table 1:** Patient demographics SD = standard deviation

	(n=83)	
	Mean ± SD	Range
Age (years)	59.3 ± 5.9	31-79
	Number	%
Sex		
Male	39	47.0
Female	44	53.0
Ethnicity		
White British	4	4.8
White European	9	10.8
Any other White background	3	3.6
Black African	10	12.0
Black Caribbean	13	15.7
Any other Black background	9	10.8
Indian	10	12.0
Pakistani	6	7.2
Chinese	4	4.8
Any other Asian background	7	8.4
Any other ethnic group	5	6.0
Not stated	3	3.6

Recurrence rate and factors related to recurrence

The different surgical procedures and the recurrences with each technique performed are presented in Table 2. Of the 83 eyes, the overall recurrence rate was 6.0%, which was observed after surgery at one, two, three, and six months in two, one, one and one eye respectively. 

**Table 2:** Different surgical procedures, frequency of recurrence and mean time of onset of recurrence CAG = conjunctival autograft; AMT = amniotic membrane transplantation

	Total Number of eyes (%)	Number of recurrences (%)	Mean time to recurrence (months)
Surgical procedure			
Primary closure	5 (6.0)	3 (60)	1.67
CAG	76 (91.6)	1 (1.3)	3
AMT	2 (2.4)	1 (50)	3
Total	83 (100)	5 (6.0)	1.96

Of the 65 eyes operated on by a consultant, recurrence was seen in four cases (6.2%). Of the 18 eyes operated on by a supervised resident, recurrence was seen in one case (5.6%). Of these 18 eyes operated on by a supervised resident, CAG was performed in 16 eyes and primary closure in the other two eyes.

When using multiple logistic regression analysis, recurrence of pterygium was not significantly associated with surgeon grade (p=0.38), gender (p=0.23) or ethnicity (p=0.17).

However, recurrence was significantly more common in patients below the age of 40 years (p=0.03). Four of five recurrences occurred in patients under the age of 40. Of these four eyes, one underwent CAG, pne underwent AMT, and two underwent primary closure. 

A summary of the cases that showed recurrence is shown in Table 3. 

**Table 3:** Recurrence cases broken down to highlight patient demographics, type of surgery, grade of surgeon and time to recurrence AMT = amniotic membrane transplantation; CAG = conjunctival autograft

Recurrence	Patient demographics			Type of surgery	Surgeon grade	Time to recurrence (months)
	Age (years)	Gender	Ethnicity			
1	35.3	Male	Indian	Primary closure	Consultant	1
2	56.5	Female	White European	AMT	Consultant	3
3	30.1	Female	Black African	Primary closure	Resident	1
4	34.2	Male	Black Caribbean	Primary closure	Consultant	3
5	36.3	Male	Any other Black background	CAG	Consultant	3

## Discussion

This retrospective study aimed to audit our results of primary pterygium excision and analyze for factors that increase the risk of recurrence. 

There are several proposed techniques for pterygium excision surgery, but the ultimate aim is to use an approach that has a low recurrence rate, minimal complications, and a good cosmetic outcome. 

Our study found that with primary closure recurrence rate was 60% (n=5). This is in keeping with many other studies that have demonstrated recurrence rates of 45-69% [15, 16]. Due to this very high recurrence rate, this approach is no longer recommended. We have very small numbers in this group as this is not our preferred surgical approach. Four of our patients opted for this approach due to patient choice. One patient was planned for CAG but converted to primary closure due to a very high systolic and diastolic blood pressure intraoperatively. 

AMT has proven useful in several ocular surface conditions such as corneal ulcers, perforations, and chemical injuries [17]. The basement membrane of the amniotic membrane promotes epithelial growth and the stromal matrix reduces scarring by suppressing myofibroblast differentiation from the normal conjunctiva, pterygium body, and corneal and fibroblasts [18]. 

We found a recurrence rate of 50% with a small sample size of two eyes. This is higher than other studies that have reported recurrence rates of 10.9% and 26.7% with AMT [16, 19]. The patient that showed recurrence in our study had both nasal and temporal pterygium in the same eye and as such the area of bare scleral left after pterygium excision was larger than normal. The higher recurrence rate of this approach in our study could be attributed to the small sample size not being representative of the pterygium seen in the population or due to inadequate excision of subconjunctival tissue. The other patient who had AMT performed that did not show recurrence opted for CAG as they wanted to have the option of trabeculectomy surgery for their glaucoma management in the future.

The use of CAG is the procedure of choice at our unit since Kenyon et al. reported a 5% recurrence rate for both advanced and recurrent pterygium and no serious complications [20]. Our study reported an overall low clinically significant recurrence rate of 1.3% for pterygium excision with CAG using fibrin glue, with a median time elapsed since surgery of 2.98 months. This is similar to the lowest recurrence rate found at 1.5% in the most recent systematic review of CAG for primary pterygium excision [10]. 

In our study, all patients had CAG fixed with glue (Tisseel) which is a fibrin sealant. The use of fibrin glue for fixing conjunctival autografts was first described in 1993 [21]. The use of glue is preferred at our unit over sutures as it has been demonstrated to produce less post-operative inflammation, greater patient comfort, and a lower recurrence rate [22, 23]. 

Our study found recurrence to be significantly higher in patients below 40 years of age (12.5% vs 3.4%, p=0.03). This reflects similar findings in other studies [24-26]. It is thought that lipoid degeneration in the peripheral cornea in more elderly patients plays an inhibitory role in pterygium recurrence and progression [27]. Other studies have found higher recurrence rates in black patients, however, we found that ethnicity was not associated with a higher recurrence rate. 

Some studies have found significantly higher recurrence rates in surgeries performed by residents compared to consultants [28, 29]. We found no difference in recurrence rate between eyes operated on by consultant or ophthalmology resident (6.1% for consultant and 5.6% for resident) which reflects what the largest prospective study showed [30]. The reasons for this may be partly due to the fact that all surgery was closely supervised by a corneal consultant in this study. Furthermore, it is important to note that only 18 eyes were operated on by a resident, and of these 16 had CAG whilst two had primary closure and due to the small numbers we cannot assess for statistical significance.

In our study, all surgeries were performed or supervised by the same surgeon which decreases the risk of technique variation. Our recurrence rate with CAG was low and equivalent to other literature reports. However, it is important to acknowledge the intrinsic limitations of our study as a retrospective series of cases. Furthermore, due to the very low sample size of eyes in the primary closure and AMT groups, we can only provide descriptive reports on these approaches and cannot provide any comparative statistical analysis between the different surgical approaches.

## Conclusions

In conclusion, this study supports the use of pterygium excision with conjunctival autograft and the fixation with Tisseel glue. There are minimal complications and a low recurrence rate that is comparable with other studies. Prospective and randomized studies with longer follow-ups are required to analyze the true effectiveness of this technique in relation to quality of vision and quality of life.
